# Facteurs pronostiques du cancer de l'œsophage au Cameroun: étude multicentrique

**DOI:** 10.11604/pamj.2019.33.73.16112

**Published:** 2019-05-31

**Authors:** Winnie Tatiana Bekolo Nga, Servais Albert Fiacre Bagnaka Eloumou, Jean Paul Ndamba Engbang, Esther Mbassi Dina Bell, Anne Marthe Maison Mayeh, Etienne Atenguena, Martin Essomba Biwole, Georges Barthélémy Nko'o Ayissi, Gabin Kenfack, Dominique Noah Noah, Henry Namme Luma, Albert Mouelle Sone, Paul Ndom, Elie Claude Ndjitoyap Ndam

**Affiliations:** 1 Service de Médecine Interne de l'Hôpital Général de Douala, Douala, Cameroun; 2 Faculté de Médecine et de Sciences Pharmaceutiques de Douala, Douala, Cameroun; 3 Service d'Oncologie et de Radiothérapie de l'Hôpital Général de Douala, Douala, Cameroun; 4 Service de Médecine et d'Oncologie de l'Hôpital Général de Yaoundé, Yaoundé, Cameroun; 5 Direction de la Lutte contre la Maladie- Ministère de la Santé Publique, Yaoundé, Cameroun; 6 Faculté de Médecine et de Sciences Biomédicales de Yaoundé, Yaoundé, Cameroun

**Keywords:** Survie, facteur pronostic, cancer de l´œsophage, Cameroun, Survival, prognostic factor, esophageal cancer, Cameroon

## Abstract

**Introduction:**

Le cancer de l'œsophage est une affection rare. Le pronostic péjoratif est lié au diagnostic tardif. La survie à 5 ans est de moins de 20%. Le but de ce travail était de rechercher les facteurs associés à la survie des patients atteints d'un cancer de l'œsophage au Cameroun.

**Méthodes:**

Il s'agissait d'une étude pronostique, sur une période de 11 ans allant du 1^er^ janvier 2005 au 31 décembre 2015 dans les Hôpitaux Généraux de Yaoundé et de Douala. Les paramètres étudiés étaient ceux associés à la survie. La survie était établie en fonction de la date du diagnostic et de la date du décès ou de la dernière consultation. Le logiciel SPSS (Statistical Package for Social Sciences) version 23 a permis l'analyse des données. La survie était présentée sous forme de courbe de Kaplan Meier. Le Test de Log Rank a permis la comparaison entre les différents groupes. La régression de Cox a permis de ressortir les différents facteurs associés. Le seuil de significativité était de 0,05.

**Résultats:**

Nous avons colligé 49 dossiers. L'âge moyen était de 57,83 ans. Le sexe masculin était présent dans 71,4% (n = 35) des cas pour un sex ratio à 2,49. Le suivi moyen était de 3,2 mois. La médiane de survie était de 6,67 mois (IC95% [1,33-10,4]) et la moyenne de survie était de 7,99 mois (IC95% [4,42-11,17]). En analyse multivariée après ajustement il ressortait que le stade IV était un facteur prédictif de mortalité (HR = 2,79; IC95% [1,13-6,89], p = 0,025]).

**Conclusion:**

Le cancer de l'œsophage reste une affection rare au pronostic péjoratif. Le facteur pronostique est le stade tumoral.

## Abstract (en)

**Introduction:**

Esophageal cancer is rare. Poor prognosis is due to delayed diagnosis. Five year survival is less than 20%. This study aimed to investigate the factors associated with survival of patients with esophageal cancer in Cameroon.

**Methods:**

We conducted a prognostic study in the General Hospitals in Yaoundé and in Douala over a period of 11 years from 1 January 2005 to 31 December 2015. The parameters studied were those associated with survival. Survival was established on the basis of the date of diagnosis and of the date of death or of the last visit. SPSS software (Statistical Package for Social Sciences) version 23 allowed for data analysis. Kaplan Meier curve was used to estimate the survival function. Log RankTest allowed for comparison among the different groups. Cox regression helped to highlight the different factors associated with it. Significance level was set at 0.05.

**Results:**

We collected data from 49 medical records. The average age of patients was 57.83 years. There was a male predominance (71.4%; n=35) with a sex ratio of 2.49. The follow-up period was 3.2 months. Median survival was 6.67 months (CI 95% [1.33-10.4]) and the average survival time was 7.99 months (CI 95% [4.42-11.17]). Multivariate-adjusted analysis showed that stage IV was a predictive factor of mortality (HR = 2.79; CI95% [1.13-6.89], p = 0.025]).

**Conclusion:**

Esophageal cancer is a rare disease with poor prognosis. Prognostic factor is the tumor stage.

## Introduction

Le cancer de l'œsophage est une pathologie grave et généralement de très mauvais pronostic [1]. Il occupe le 4^ème^ rang des cancers digestifs, en termes d'incidence dans le monde [1]. En 2012, plus de 456.000 nouveaux cas ont été enregistrés dans le monde, dont 27.500 en Afrique [1, 2]. La répartition varie sur le plan géographique [1, 2]. Ainsi 89% des nouveaux cas enregistrés dans le monde, vivaient dans les pays en voie de développement, et en Afrique 80% étaient en Afrique subsaharienne 2012 [1, 2]. L'hétérogénéité observée dans la répartition géographique serait en rapport avec les facteurs de risque, qui sont variables d'une région à une autre [1, 2]. De manière générale, les principaux facteurs de risque sont le tabac, l'alcool, le reflux gastro-œsophagien et les lésions précancéreuses comme l'endobrachyœsophage [1, 3]. Dans les pays en voie de développement, et notamment en Afrique, une alimentation pauvre en oligo-éléments mais aussi les infections notamment à human papilloma virus, semblent être des facteurs de risque additionnels [2, 4, 5]. Ces différents facteurs de risque influent également sur le type histologique. Le carcinome épidermoïde qui est le type le plus fréquent est en rapport avec la consommation d'alcool et de tabac, tandis que l'adénocarcinome est lié au reflux gastro-œsophagien et à un œsophage de Barett [2, 5]. Le cancer de l'œsophage occupe la 6^ème^ place de tous les cancers en termes de décès, ce qui en fait l'un des cancers les plus agressifs [1, 2]. Cette mortalité est étroitement liée à l'incidence, la majorité des cas se trouvant en Asie et en Afrique subsaharienne [1, 2]. En Afrique en 2012, on a enregistré 25200 décès liés au cancer de l'œsophage [2]. La survie à 5 ans est estimée entre 13-20% selon les études, qui concernent le plus souvent des séries occidentales ou asiatiques [1, 5-7]. Ces études ont également permis de déterminer les différents facteurs pronostiques, qui sont l'âge, le sexe, la race et le stade tumoral [5-7]. Très peu d'étude sur la survie ont été réalisées en Afrique en général et au Cameroun en particulier. Le dernier travail sur le cancer de l'œsophage au Cameroun portait sur les aspects anatomopathologiques et cliniques [8]. Le but de ce travail était de rechercher les facteurs prédictifs de mortalité chez le patient camerounais porteur du cancer de l'œsophage.

## Méthodes

**Type, durée et lieu de l'étude:** nous avons mené une étude transversale et analytique. Elle s'étalait sur une période de 11 ans allant du 1^er^ janvier 2005 au 31 décembre 2015. Le cadre était les services d'oncologie et de médecine interne des Hôpitaux Généraux de Yaoundé et de Douala. Les Hôpitaux Généraux de Yaoundé et Douala sont des hôpitaux de références avec une fonction universitaire. Ils sont classés dans la première catégorie de la pyramide sanitaire du Cameroun. La capacité respective est de 302 lits pour Yaoundé et 320 lits pour Douala. Ce sont les deux plus grands centres d'oncologie au Cameroun.

**Population d'étude:** la population étudiée était celle des patients atteints d'un cancer de l'œsophage. Étaient inclus dans ce travail tout patient porteur d'un cancer de l'œsophage confirmé par un résultat histologique. Etait exclu tout patient porteur d'un cancer ORL synchrone ou de la jonction œsogastrique.

**Données recueillies:** les données recueillies étaient collectées à partir des informations retrouvées dans les dossiers des patients. Les paramètres étudiés étaient sociodémographiques (âge, sexe, domicile et le mode de paiement), comorbidités, facteurs de risque, cliniques (date du diagnostic, date de décès, date de la dernière consultation et symptômes), paracliniques (endoscopie, anatomie pathologique), stade tumoral, thérapeutique, traitement et suivi (décès, rémission, perdu de vue). La présentation endoscopique était d'une part faite en fonction de la localisation de la tumeur: tiers supérieur, moyen, inferieur ou encore mixte et d'autre part de l'aspect endoscopique: ulcéré, ulcéro-bourgeonnant, sténosant. Le stade tumoral était défini en fonction de la classification pTNM de l'AJCC 7^ème^ édition [9]. Sur le plan thérapeutique, nous avons séparé les patients en deux catégories, d'une part ceux qui n'avaient pas reçu de traitement et d'autre part ceux qui en avaient reçu. Pour les patients qui avaient reçu un traitement, nous avons noté le type de traitement reçu (chirurgie, chimiothérapie, radiothérapie et/ou radiochimiothérapie). La rémission était définie ici comme l'absence de progression tumorale pendant la durée de suivi.

**La survie et pronostic:** la survie était calculée en fonction de la date du diagnostic et celle de la dernière consultation ou du décès. L'évaluation de la survie était faite à un, trois, neuf et douze mois. La durée de suivi était calculée en fonction de la date de première et de la dernière consultation. Nous avons ressorti la mortalité en fonction de la population de cette étude.

**Analyse statistique:** la collection et l'analyse des données ont été faites par les logiciels Microsoft Excel 2013 et IBM SPSS (Statistical Package for Social Sciences) version 23. Les graphiques ont été faits à l'aide du logiciel GraphPad Prism Version 6.01. Les variables qualitatives sont présentées sous forme de proportion et de fréquence et les variables quantitatives sous forme de moyenne avec écart type ou de médiane avec Intervalle Interquartile (IIQ). Le test de log Rank a permis de comparer les différents groupes. La survie a été présentée sous forme de courbe de Kaplan Meier. Le modèle de régression de Cox a permis après analyse uni et multivariée de rechercher les facteurs prédictif de mortalité par un Hazard Ratio (HR). Le seuil de significativité était inférieur à 0,05.

**Considération éthique:** ce travail a reçu sur le plan éthique une clairance éthique des comités institutionnels de chacun des hôpitaux généraux de Yaoundé et Douala.

## Résultats

### Caractéristiques générales de la population d'étude

Nous avons colligé 49 patients porteurs d'un cancer de l'œsophage pendant la période l'étude. La moyenne d'âge était de 5^7^,84 ± 12,6 ans (Tableau 1). Le sexe masculin était présent dans 71,4% (n = 35) des cas avec un sex ratio à 2,49 en faveur des hommes (Tableau 1). La population vivant en zone urbaine était la plus fréquente avec 73,5% (n = 36) des cas (Tableau 1). Le paiement des frais de consultation était à la charge du patient dans la majorité des cas (95,99% soit 47 patients (Tableau 1). La consommation d'alcool et celle du tabac était respectivement à 59,2% (n = 29) et 44,9% (n = 22) des cas (Tableau 1). Aucun patient n'avait un antécédent familial de cancer ORL ou de l'œsophage. Les symptômes les fréquents étaient la dysphagie aux solides, l'amaigrissement, la dysphagie aux liquides et la douleur épigastrique et retrouvé respectivement chez 87,8% (n = 43), 75,5% (n = 37), 38,8%(n = 19) et 36,7%(n = 18) des cas. L'ensemble des patients présentait au moins un symptôme. Sur le plan endoscopique, la principale localisation de la tumeur était le tiers inférieur, observée dans 44,9% (n = 22) des cas (Tableau 1). La lésion retrouvée était le plus souvent ulcéro-bourgeonnante dans 42,9% (n = 21) des cas (Tableau 1). Le carcinome épidermoïde était la forme histologique observée chez 73,5% (n = 36) des cas (Tableau 1). Le stade tumoral IV était retrouvé dans 34,7% (n = 17) des cas (Tableau 1). Les données sur le traitement ont été consignées dans le Tableau 1. La majorité des patients soit 79,6% (n = 39) des cas avait reçu un traitement. La chirurgie avait été réalisée chez 10 patients, la chimiothérapie chez 34 patients et la radiothérapie chez 15 patients. La rémission après un des traitements suscités était observée chez 5 patients soit 10,4% des cas.

**Tableau 1:** Caractéristiques de la population d’étude

Paramètres	Valeur n (%)
Sexe (masculin)	35(71,4)
Domicile (urbain)	36(73,5)
Mode de paiement (espèce)	47(95,9)
Consommation d’alcool	29(29,2)
Prise de tabac	22(44,9)
Dysphagie aux solides	43(87,8)
Amaigrissement	37(75,5)
Dysphagie aux liquides	19(38,8)
Siège de la tumeur (tiers inferieur)	22(44,9)
Aspect endoscopique (ulcéro-bourgeonnant)	21(42,9)
Nature histologique (carcinome épidermoïde)	26(73,5)
Degré de différentiation tumoral (moyennement différencié)	20(40,8)
Stade tumoral (stade IV)	15(30,6)
Patients traités	39(79,6)
Décès	24(50,0)
Rémission	5(10,4)

**Tableau 2:** Facteurs prédictifs de mortalité de la population de l’étude

Variables	HR (IC à 95%) Univariée	p	HR ajusté (IC à 95%) Multivariée	p ajustée
**Stade tumoral**				
IV	**5,2 (1,8-14,4)**	**0,002**	**2,79 (1,13-6,89)**	**0,025**
Non IV	0,19 (0,07-0,54)	0,002	0,36 (0,15-0,88)	0,025
**Consommation d’alcool**				
Oui	2,04 (0,82-4,89)	0,082	/	/
Non	0,49 (0,2-1,17)	0,082		
**Aphagie**				
Oui	20,55 (2,08-203)	0,009	2,28 (0,59-8,84)	0,232
Non	0,05 (0,005-0,48)	0,009	0,44 (0,11-1,69)	0,025

### Survie et facteurs prédictifs de mortalité

**Figure 1:**
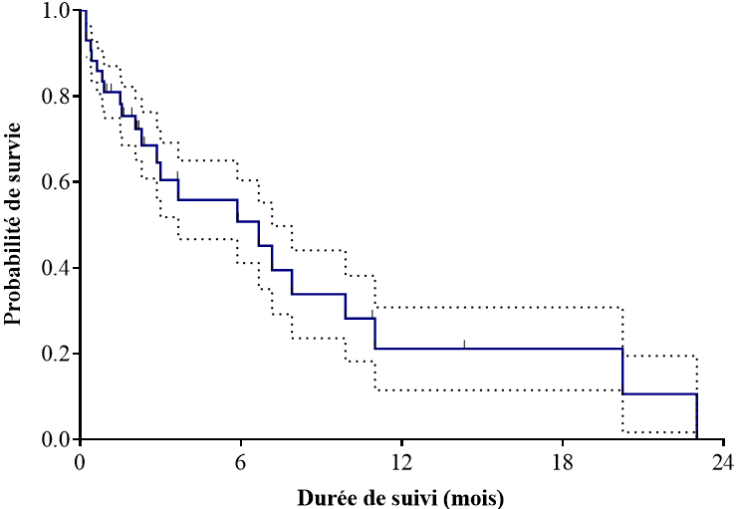
Survie globale de la population d'étude

**Figure 2:**
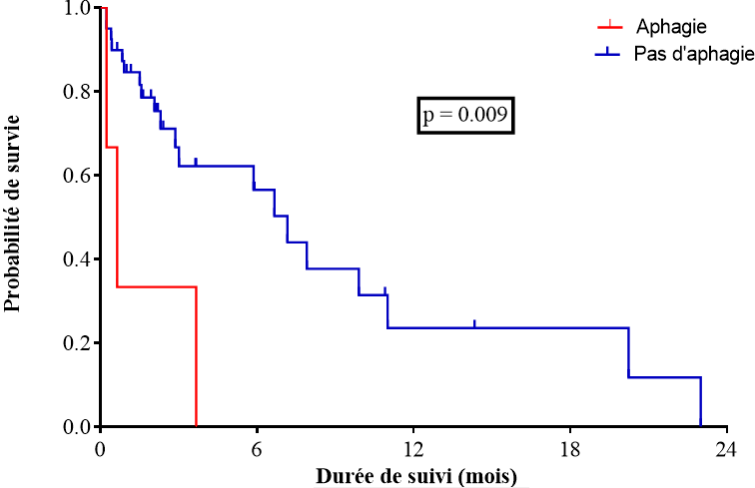
Survie de la population d'étude en fonction de l'aphagie

**Figure 3:**
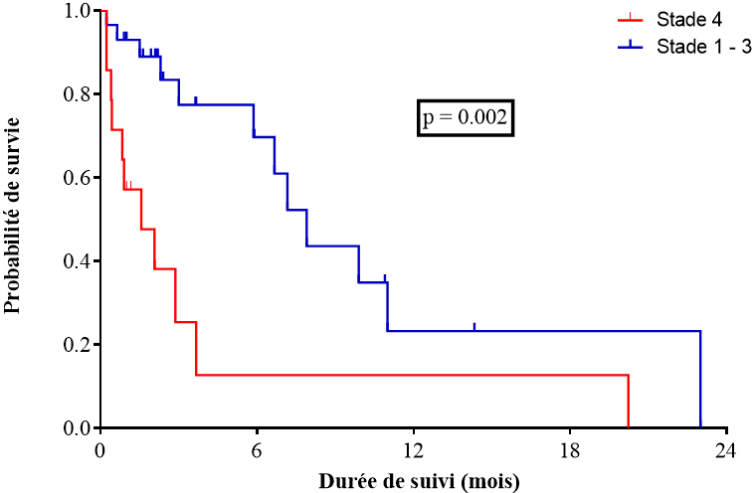
Survie de la population d'étude en fonction du stade tumoral

Il ressort dans ce travail que la mortalité globale dans la population étudiée était de 50%. Le délai moyen entre le début des symptômes et le diagnostic était de 137 ± 118 jours. Il y avait 19 patients perdu de vue pendant le suivi. Cette survie était en moyenne de 3,2 mois. La médiane de survie était de 6,67 mois (IC95% [1,33-10,4]) et la moyenne de survie était de 7,99 mois (IC95% IC95% [4,42-11,17]). La survie à 3, 6, 9 et 12 mois était respectivement de 60,5%, 50,8%, 28,2% et 21,1% (Figure 1). En analyse univariée les facteurs prédictifs de mortalité étaient la présence d'une aphagie (HR = 20,55; IC95% [2,08-203]; p = 0,009) (Figure 2), le stade IV (HR = 5,2; IC95% [1,8-14,4,] p = 0,002) (Figure 3). En analyse multivariée après ajustement il ressortait que le stade IV était un facteur prédictif de mortalité (HRa = 2,79; IC95% [1,13-6,89], p = 0,025]) (Tableau 2).

## Discussion

L'analyse des résultats obtenus, nous montre une certaine constance sur le plan épidémiologique par rapport à ceux retrouvés il y a près de vingt-cinq ans par Mbakop *et al* [8]. Le faible nombre de patients colligés dans notre étude, ceci par rapport à la période de notre étude confirme que le cancer de l'œsophage au Cameroun est une pathologie rare, bien qu'il ne reflète pas l'incidence réelle de cette pathologie dans le pays. Ce nombre est d'autant plus faible lorsqu'il est comparé à celui des séries d'Afrique de l'Est, d'Europe ou encore d’Asie, qui sont des zones de fortes endémicités [1, 3, 10, 11]. Bien que l'âge de nos patients soit légèrement supérieur à bon nombre de séries africaines, il est semblable à celui retrouvé en Chine. Il reste tout de même nettement inférieur à celui des patients en Europe ou aux États-Unis, où il se situe entre 60-70 ans [3]. En Afrique et même en chine, l'existence de double cohorte avec deux groupes d'âge, l'un avec des patients très jeunes peut souvent expliquer le jeune âge des patients [12-14]. La prédominance masculine du cancer de l'œsophage que nous avons retrouvé, est commune quelle que soit la zone géographique [1-3]. Toutefois, on observe une augmentation de l'incidence du cancer chez les femmes, et dans certaines régions en Iran ou encore en Chine, le sex ratio est de 1:1 [5]. Sur le plan histologique, le carcinome épidermoïde comme dans le reste du monde surtout dans les pays d'Asie et en Afrique est le type histologique prédominant au Cameroun [1, 3, 5, 8]. Ce résultat reste à relativiser lorsqu'on voit la dynamique d'évolution du type histologique dans le monde avec une nette augmentation de l'incidence de l'adénocarcinome [15]. Ainsi les différentes projections en 2030, montrent qu'en Europe de l'ouest et même en Australie, l'adénocarcinome deviendrait le type histologique le plus fréquent, ce qui n'est pas le cas aux États-Unis et ce, quel que soit la race [15]. Au moment du diagnostic, la majorité de nos patients ont une tumeur localement avancée avec des métastases, car le délai entre le début des symptômes et le diagnostic était très long. Le stade tumoral avancé est commun aux pays en voie de développement et même en Europe. En Asie notamment en Chine et au Japon les campagnes de dépistage réduisent considérablement ces délais ce qui rend le diagnostic plus précoce [6, 16].

De manière générale, la survie qui est de 6.67 mois, est très faible. Le taux de survie élevé à 3 mois peut être corrélé à la durée moyenne du suivi de nos patients. Il a tendance à diminuer au fil des mois. La survie de nos patients est semblable à celle de Mmbaga *et al*. en Tanzanie [17]. Elle reste bien en deçà, de celle retrouvée par Zhang *et al*. où elle était de 15,3 mois dans la population chinoise et de 14,2mois dans la population caucasienne [6]. Ces chiffres confirment le mauvais pronostic du cancer de l'œsophage, où la survie à 5 ans est généralement de moins de 20% [1, 3, 5, 6]. Ce pronostic est meilleur en Europe et en Asie à cause d'une part du niveau socio-économique et d'autre part du dépistage systématique. Plusieurs facteurs pouvant modifier la survie ont été retrouvés, à savoir l'âge des patients, le sexe, la race, le niveau socio-économique bas, la nutrition, la prise en charge des complications liées à la maladie [2, 3, 7, 18, 19]. Le facteur qui semble être commun à toutes ses études mais aussi à la nôtre est le stade tumoral, car il détermine le type de traitement. Les meilleurs taux de survie ont été obtenus à des stades plus ou moins précoces [3, 20].

## Conclusion

Le cancer de l'œsophage reste une pathologie rare au Cameroun et de très mauvais pronostic. Le diagnostic est fait le plus souvent à un stade avancé et la survie est très faible. Le stade tumoral IV est associé de façon indépendante à la survie. Toutefois la mise sur pied d'un registre national du cancer nous permettrait d'avoir plus d'informations tant sur l'épidémiologie mais aussi les facteurs pronostiques du cancer au Cameroun.

### Etat des connaissances actuelles sur le sujet

- Le cancer de l'œsophage occupe le 4^ème^ rang des cancers digestifs, en termes d'incidence dans le monde;
- L'Afrique subsaharienne est la zone la plus touchée, avec 89% des cas;
- Le cancer de l'œsophage est une pathologie grave et généralement de très mauvais pronostic.

### Contribution de notre étude à la connaissance

- Ce travail a permis ainsi d'apporter des données récentes sur le cancer de l'œsophage au Cameroun, répondant ainsi au manquement sur la question;
- Ce travail a aussi permis de déterminer les facteurs pronostiques de cette pathologie;
- Ce travail ouvre le débat sur la nécessité d'un registre des cancers et de la prévention dans les pays à ressources limitées.

## Conflits d’intérêts

Les auteurs ne déclarent aucun conflit d’intérêts.

## Remerciements

Les auteurs remercient le personnel paramédical des Services de Médecine interne et d'Oncologie des Hôpitaux Généraux de Douala et Yaoundé pour l'aide apportée dans la collecte des données.

## References

[1] Ferlay J, Soerjomataram I, Dikshit R, Eser S, Mathers C, Rebelo M (2015). Cancer incidence and mortality worldwide: sources, methods and major patterns in GLOBOCAN 2012. Int J Cancer.

[2] Parkin DM, Bray F, Ferlay J, Jemal A (2012). Cancer in Africa. Cancer epidemiology, biomarkers & prevention: a publication of the American Association for Cancer Research, cosponsored by the American Society of Preventive Oncology.

[3] Zhang Y (2013). Epidemiology of oesophagal cancer. World J of Gastroenterol.

[4] Ludmir EB, Stephens SJ, Palta M, Willet CG, Czito BG (2015). Human papillomavirus tumor infection in esophageal squamous cell carcinoma. J Gastrointest Oncol.

[5] Mao WM, Zheng WH, Ling ZQ (2011). Epidemiologic risk factors for esophageal cancer development. Asian Pac J Cancer Prev.

[6] Zhang J, Jiang Y, Wu C, Cai S, Rui Wang, Ying Zhen (2015). Comparison of clinicopathologic features and survival between eastern and western population with esophageal squamous cell carcinoma. J Thorac Dis.

[7] Napier KJ, Scheerer M, Subhasis M (2014). Esophageal cancer: A Review of epidemiology, pathogenesis, staging workup and treatment modalities. World J Gastrointest Oncol.

[8] Mbakop A, Ndjitoyap Ndam EC, Biwole M, Tagny Sartre M, Tchouanhou I, Michel G (1990). Cancers de l'œsophage en milieu camerounais: aspects anatomo-pathologiques et cliniques. Revue médicale de Côte d'Ivoire.

[9] Rice TW, Blackstone EH, Rusch VW (2010). 7th edition of the AJCC Cancer Staging Manual: esophagus and eosophagogastric junction. Ann Surg Oncol.

[10] Wakhisi J, Patel K, Buziba N, Rotich J (2005). Esophageal cancer in north rift valley of Western Kenya. Afr Health Sci.

[11] Kashala R (2010). Systematic review: epidemiology of Oesophageal Cancer in SubSaharan Africa. Malawi Medical Journal.

[12] Ndiaye B, Ndiaye AR, Gning SB, Diop Y, Diagne M, Fall AK (2009). Le cancer de l'œsophage au Sénégal: une double population. Gastroenterol Clin Biol.

[13] Parker RK, Dawsey SM, Abnet CC, White RE (2010). Frequent occurrence of esophageal cancer in young people in western Kenya. Dis Esophagus.

[14] Chen W, He Y, Zheng R, Zhang S, Zeng H, Zou X (2013). Esophageal cancer incidence and mortality in China, 2009. J Thorac Dis.

[15] Arnold M, Laversanne M, Morris Brown L, Devesa SS, Bray F (2017). Predicting the Future Burden of Esophageal Cancer by Histological Subtype: International Trends in Incidence up to 2030. Am J Gastroenterol.

[16] Lin Y, Totsuka Y, He Y, Kikuchi S, Qiao Y, Ueda J (2013). Epidemiology of esophageal cancer in Japan and China. J Epidemiol.

[17] Mmbaga EJ, Deardorff P, Mushi B, William Mgisha, Megan Merritt, Robert A Hiatt (2018). Characteristics of Esophageal Cancer Cases in Tanzania. J Glob Oncol.

[18] Baquet CR, Commiskey P, Mack K, Meltzer S, Mishra SI (2005). Esophageal cancer epidemiology in blacks and whites: racial and gender disparities in incidence, mortality, survival rates and histology. J Natl Med Assoc.

[19] Matsumoto H, Okamoto Y, Kawai A, Ueno D, Kubota H, Murakami H, Higashida M, Hirai T (2017). Prognosis Prediction for Postoperative Esophageal Cancer Patients Using Onodera's Prognostic Nutritional Index. Nutr Cancer.

[20] Kjaer DW, Larsson H, Svendsen LB, Jensen LS1 (2017). Changes in treatment and outcome of oesophageal cancer in Denmark between 2004 and 2013. Br J Surg.

